# Hurdles and delays in access to anti-cancer drugs in Europe

**DOI:** 10.3332/ecancer.2014.482

**Published:** 2014-11-17

**Authors:** F Ades, D Zardavas, C Senterre, E de Azambuja, A Eniu, R Popescu, M Piccart, F Parent

**Affiliations:** 1 Department of Medical Oncology, Institut Jules Bordet, Université Libre de Bruxelles, Brussels 1000, Belgium; 2 Research Centre of Epidemiology, Biostatistics and Clinical Research, School of Public Health, Université Libre de Bruxelles, Brussels 1050, Belgium; 3 Department of Breast Tumours, Cancer institute Ion Chiricuta, Cluj-Napoca RO-400015, Romania; 4 Department of Medical Oncology, Hirslanden Clinic Aarau, Aarau 5001, Switzerland; 5 Research Centre of Social Approaches of Health, School of Public Health, Université Libre de Bruxelles, Brussels 1050, Belgium

**Keywords:** drug uptake, European Medicines Agency (EMA), European Union, marketing authorization, pricing and reimbursement, prescription and compliance

## Abstract

Demographic changes in the world population will cause a significant increase in the number of new cases of cancer. To handle this challenge, societies will need to adapt how they approach cancer prevention and treatment, with changes to the development and uptake of innovative anticancer drugs playing an important role. However, there are obstacles to implementing innovative drugs in clinical practice. Prior to being incorporated into daily practice, the drug must obtain regulatory and reimbursement approval, succeed in changing the prescription habits of physicians, and ultimately gain the compliance of individual patients. Developing an anticancer drug and bringing it into clinical practice is, therefore, a lengthy and complex process involving multiple partners in several areas. To optimize patient treatment and increase the likelihood of implementing health innovation, it is essential to have an overview of the full process. This review aims to describe the process and discuss the hurdles arising at each step.

## Introduction

Cancer is a major public health problem in both developed and developing countries. Europe reported around 3.5 million new cases of cancer in 2008, a number expected to sharply increase given the significant ageing of European population [1]. Moreover, for a number of cancers, the current therapeutic options are unable to control the evolution of the disease. Therefore, the development and uptake of innovative anticancer drugs is one of the most critical aspects of addressing this problem.

**Figure 1.**
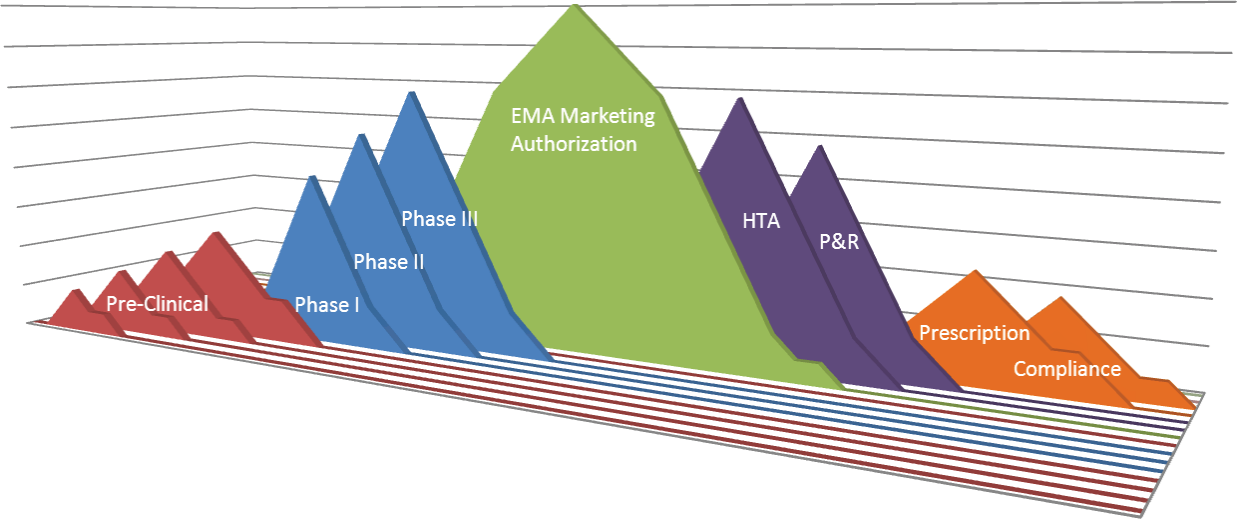
Drug hurdles: from drug target identification to patient compliance—the long way from the bench to the patient. EMA: European Medicines Agency; HTA: health technology assessment; P&R: pricing and reimbursement.

Medical innovation has a major impact on patient survival and longevity. Econometric approaches estimate that 40% of the improvement in life expectancy in recent years was related to the launch of innovative drugs [2]. Developing a drug and bringing it into clinical practice is a complex process involving multiple partners in several areas, including researchers, pharmaceutical companies, national governments, health authorities, the medical community, and patients. Several hurdles can arise during each stage of this process, i.e., during the drug development, the granting of access to the drug by regulators, and the adoption of the drug in clinical practice. An overview of the regulatory process to final administration to patients is fundamental to optimizing patient treatment and increasing the likelihood of implementing health innovation (Figure 1).

## The European Union centralized procedure for marketing authorization

Since 1995, the European Medicines Agency (EMA) has adopted the ‘centralized procedure’ [Regulation (EC) No 726/2004] [3] to grant marketing authorizations across all its member states. The Committee for Medicinal Products for Human Use (CHMP) [4] is the EMA board responsible for this task. It is composed of specialists nominated by each European Union (EU) member state and evaluates the safety, efficacy, and quality of medicinal products [5]. Since 2005, applications through the centralized procedure are compulsory for cancer drugs [6, 7].

### EMA ‘no-go’ decision

The drug approval rate has been relatively constant since implementing the centralized procedure: between 1997 and 2001 EMA approved 71% of all classes of drug applications [30], and in the period between 2006 and 2011, 73% were approved [8]. When restricted to cancer drugs, the approval rate is lower, around 63%. This result is largely driven by the high failure rate of biological agents in comparison to chemically synthesized drugs (33% versus 74%, *P* = 0.018 [odds ratio 0.18, 95% CI 0.04–0.75]) [8]. The leading causes of objections to applications are related to safety and adverse events (48.6%) and clinical efficacy; the absence of a randomized trial was the reason for objections in 42.3% of applications [9].

### EMA-related delays

**Figure 2.**
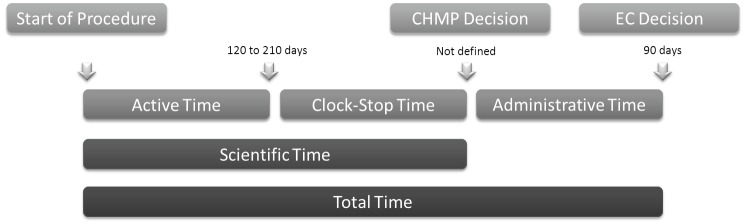
EMA timetable for evaluation of drug application through the centralized procedure.

The EMA procedure to evaluate drug approval applications through the centralized procedure consists of the following phases (Figure 2):

- ‘Active time’—when the CHMP evaluates the application. This period should last up to 210 days under the standard centralized procedure and 120 days following the accelerated evaluation procedure.
- ‘Clock stop time’ is the time needed by the applicant to answer the queries raised by the CHMP. The sum of these two times is defined as the ‘scientific time’. After this period, the CHMP decides for approval or refusal of drug application.
- ‘Administrative time’—when the European Commission evaluates the compliance of marketing authorization applications to European laws. This should last up to 90 days. The sum of these three times is defined as ‘total time’.

Anticancer drug applications generally fall within the expected timelines. Between 1995 and 2004, 20 anticancer drugs were approved. The active time evaluation in fewer than 210 days was achieved in 75% of cases, with the mean stopping time being 119 days. The administrative time ranged from 92 to 173 days, with a mean time of 117 days, slightly above the proposed 90-day target [10].

Several studies comparing the approval processes of different agencies discussed improving the effectiveness and the timeliness of such evaluations [11–14]. In 2004, the US Food and Drugs Administration (FDA) had approval times of 387 days for standard drugs and 180 days for priority drugs. In general, patients in the United States get access to new products earlier, most likely due to the frequent use of expedited review procedures [8]. In Canada, the median evaluation time was 426 days, and in Australia 321 days [10]. In Japan, times have been decreasing: in 1998, it was 1,239 days, decreasing to 531 days in 2002 [15, 16]. The medium time for drug approval in the EU is 418 days [17].

## Health technology assessment

Health technology assessment (HTA) evaluation involves the study of the medical, social, ethical, and economic implications of the development, distribution, and use of a health technology, including cost-effectiveness analysis [18]. These evaluations are tailored for health policy stakeholders and intended to bridge the gap between research data and health policy decision making.

In 2004, the European Commission targeted HTA as a priority and created the European network for Health Technology Assessment (EUnetHTA) [19], representing 63 partners from 32 countries, of which 25 are EU member states [20]. Its objectives include facilitating efficient resource use and avoiding effort overlap and duplication. The EUnetHTA works in collaboration with other international HTA organizations, with professional societies, such as the European Society for Medical Oncology (ESMO), and with the WHO and EMA [20].

HTA reports are descriptive but can also make recommendations for drug use. They are compiled in several databases, such as INATHA [21], ISPOR [22], and HEED [23].The number of cancer HTA reports has increased over the years, with breast cancer contributing to the majority of them. The United Kingdom (UK) is the most active country in producing cancer drugs health economic evaluations, issuing one-third of all reports. The National Institute for Health and Care Excellence (NICE) is an HTA agency established in 1999 proving guidance to the UK National Health System (NHS) [24]. A positive recommendation by NICE has direct effect on the allocation of budget for drug reimbursement in the UK. However, while a positive recommendation increases the uptake of a new drug, it can also create a significant lag if HTA evaluations are not undertaken in a timely manner. There is a tradeoff between access to a well-performed HTA report and the speed of uptake of new drugs.

The usefulness of HTA evaluations and guidance varies from country to country and evidence of a direct impact of HTA is not homogeneously observed [25]. Nevertheless, given the increased collaboration between regulatory agencies, it is expected that HTA importance will increase.

## Price negotiation and budget allocation

In Europe, decisions about the price of drugs paid by the health-care systems must be made before the drug is launched in the market, with the objective of controlling health budgets. This is a competency of each national government and reimbursement policies are diverse across the EU. According to Directive 89/105/EEC52, governments have 120 days to perform this task, but most decisions take longer [26].

Anticancer drugs are estimated to represent less than 15% of the total cost of oncology treatment, and around 5% of the total drug expenditure [17]. Despite this, they are a readily identified area for budget cuts, and this probably contributes most to the disparity of drug uptake across the EU [28]. Between 1998 and 2007, the sales of anticancer drugs increased from €4.3 to €26.3 per capita, a figure slightly higher than the increase of the overall cancer care cost. The expenditure per capita of all medicines in the EU was €430 per citizen [26]. Most of the increase is related to drugs approved before 1999, highlighting the substantial increase in uptake after patent expire and more affordable generics become available [18].

### Pricing

Drug production costs are generally low; however, the final cost to the buyer covers expenses associated with the research and development phases related to that drug as well as to others that fail to reach the market. In general, although not transparent, pricing policies consider three factors: (1) production costs, (2) reimbursement level, and (3) restrictions on prescription. One other important characteristic is that the commercial price of a drug does not necessarily correlate with the level of benefit offered to the patient.

Most governments control the initial prices of reimbursed medicines. They are often the biggest buyer, which allows price negotiation, establishing reference prices, reimbursement levels, and restrictions. Countries such as Denmark, Germany, Malta, Sweden, and the UK allow companies to freely determine initial drug launch prices. However, this does not mean that these countries do not have control over prices, as paybacks and price-volume agreements can be made. Other countries—e.g., Belgium, the Netherlands, Portugal, and the Nordic countries—have formalized decision-making processes requiring economic and cost-effectiveness evaluation. Another practice to control cost is sharing it with patients. Co-payments make the patient cost-sensitive and add another layer of control at the consumer end.

A common pricing practice is to compare the requested prices with the prices in other countries. As a consequence, the prices initially set serve as references for subsequent ones. This adds substantial delay to drug uptake in smaller countries given that companies generally first apply for reimbursement in bigger markets, like the UK and Germany, where prices are often freely established. In this way, initial prices are commonly set at high levels, increasing the delay and reducing the uptake of drugs in less-wealthy countries.

### Reimbursement decisions and drug lags

Delays in market access for drugs are extremely heterogeneous in Europe and depend on the country and the setting where the drugs are used. Theoretically, Germany and the UK have no reimbursement delays after EMA approval; nevertheless, drug uptake in the UK can change dramatically in the function of evaluation by NICE. Other countries have substantial delays due to formal reimbursement procedures like France, Belgium, and Italy [26]. In Romania, no new drugs have been included on the reimbursement list over the last 5 years. In general, drugs used in the hospital setting are available sooner than drugs used in the outpatient setting because, in many European countries, cancer drugs used in hospitals are paid out of the hospital budget, while drugs used in ambulatory care require formal reimbursement decisions. Using a different strategy, France, Germany, and Denmark allocate specific budgets to make innovative cancer drugs readily available [26].

**Figure 3.**
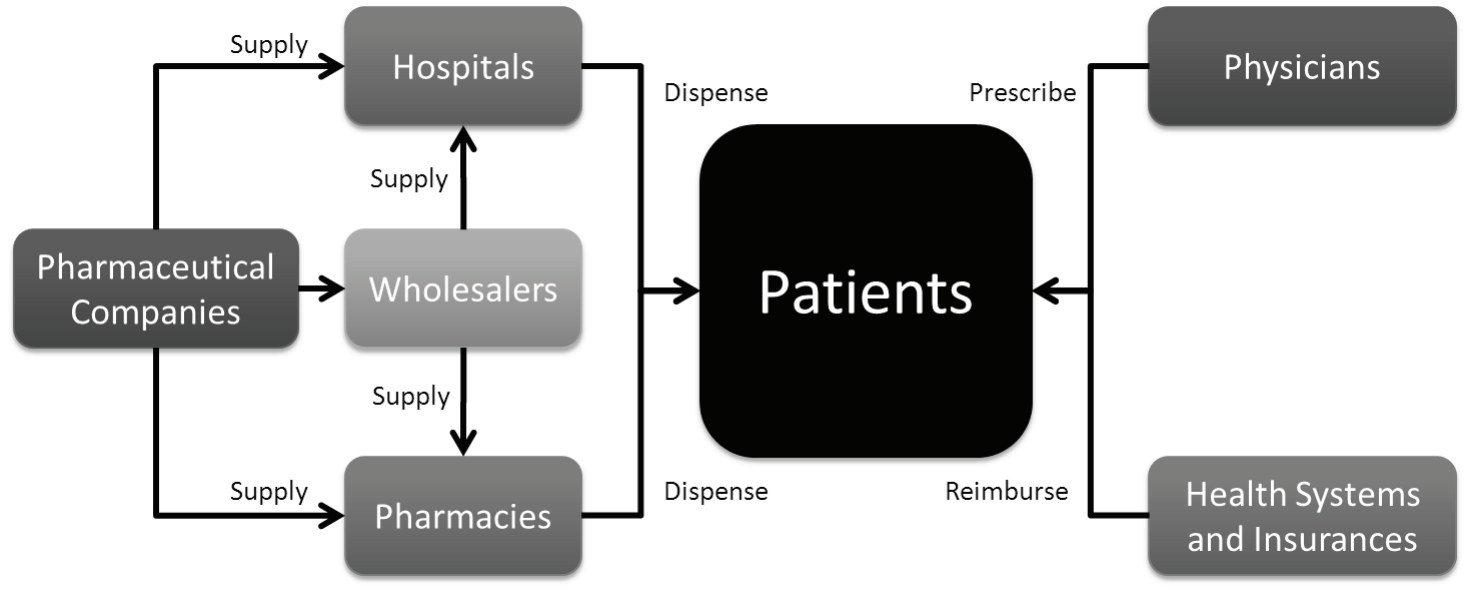
Drug flux from the pharmaceutical company to the patient.

The uptake level of any drug is also closely linked to the percentage of governmental reimbursement and patient co-payment. In the case of no-reimbursement, competition, or significant patient co-payment, uptake is generally lower. Figure 3 depicts the flow of drugs from pharmaceutical companies to the patient.

Reimbursement decisions are taken based on the expected societal benefit of a drug in relation to its costs, and many approaches are used to measure it. Health gains can be estimated in relation to the drug’s monetary value, normally evaluated as money per quality-adjusted life year (QALY, a product of the quality and the quantity of life gained with a given intervention) [29]. These analyses are context-sensitive, meaning that different countries can adopt distinct health policies and reimbursement decisions using similar results.

The UK’s NICE is among the HTA agencies that advocate for the use of QALY in health policy decision making. NICE considers cost-effective interventions costing between £18,000 and £40,000 per QALY [30], while in the Netherlands this value is around €18,000 [18]. Controversy exists in the use of QALY; while most Northern European countries are influenced by NICE’s decisions, in Southern and Eastern Europe QALY evaluations have little impact. In Germany, its evaluation has been banned [31]. In the United States, the use of QALY has been prohibited by the Patient Protection and Affordable Care Act 2010 [32].

The WHO proposes that interventions costing less than three times the GDP per capita for each disability-adjusted life year (DALY, meaning the sum of years living with disability with years of life lost) saved should be considered cost-effective [18], but no formal recommendation exists.

Italy has implemented a pivotal health policy founded on performance-based agreements conditional on clinical evaluation of specific endpoints. This is a model of sharing responsibility and risk between the public health system and the pharmaceutical companies. It happens in three ways: (1) cost sharing, consisting on discount on initial price of treatment for all patients; (2) risk sharing, when prices discounts are applicable to initial therapy cycles of non-responder patients; and (3) payment by results, when initial cycles are fully reimbursed by the pharmaceutical company if no effect is observed [33].

## Gaps and divergences between EMA evaluation and HTA appraisals and national governments’ reimbursement decisions

The mechanisms of drug marketing authorization and HTA appraisals are known and their reports publicly available. To certain degree, pricing and reimbursement negotiation decisions are also known. Nevertheless, the results of these processes do not necessarily reach a consensus. This happens mostly because different bodies evaluate different characteristics of the drug in question. In one hand EMA [34], and other regulatory bodies like the US FDA [35], focus on the evaluation of a drug clinical benefit and safety without a clear required threshold for the magnitude of benefit. In the other hand payers and HTA bodies value the relative efficacy/effectiveness of new drugs in relation to the currently approved options as well as its cost-effectiveness [36].

While some advocate for the use of QALY to be used in the marketing authorization processes [35] controversy exists in their use for policy making. As previously mentioned, in the Unites States, the Patient Protection and Affordable Care Act of 2010 specifically prohibits using cost-effectiveness measures to determine coverage, reimbursement, or incentive programmes [37].

Health costs are nevertheless to be taken in consideration in that setting. A North American study found that medical problems contributed to at least 46.2% of all bankruptcies in the United States. And this number increased to 49.6% between 2001 and 2007 [38]. Discussions for the incorporation of cost-effectiveness analyses in the North American Medicare decisions about coverage and reimbursement are still ongoing [39]. In Europe, in the face of rising prescription drug costs, price-control measures are in place in several countries [40].

One pilot policy in pricing was established in Germany, which enacted the Act on the Reform of the Market for Medical Products (AMNOG). This is an innovative approach to assess additional benefits of new drugs launched in the German market and pricing according to their efficacy. After 1 year of its use, if no additional benefit is found, the pharmaceutical is allocated to a reference price group with comparable active ingredients. If an additional benefit is observed, a supplement on top of the price of the expedient comparative therapy is granted [41].

In the United States, where drug costs are paid by the health insurance companies and not the national government, strategies adapted to this health system are being tested. Current cancer guidelines often present a list of all possible or acceptable treatment options, but they do not provide comparative analysis to enable patients and physicians to choose the most cost-effective option [35]. The Drug Effectiveness Review Project is an initiative proposing that, after approval of a drug, a population-based database would be established. This information would allow for comparisons across different drugs and give patients and physicians the ability to make better informed decisions about treatments [42].

### The adaptive licensing approach

Marketing authorization decisions are dichotomously taken (granted versus not granted) based on a review of safety and efficacy data. Overall survival is considered the gold standard trial end point for marketing approval; nonetheless, additional and surrogate endpoints have been used. The idea is to accelerate approval, with a sponsor commitment to providing evidence of clinical benefit in subsequent trials [43].

The adaptive licensing approach is a different strategy to improve timely access for patients to new medicines. It is a prospectively planned process, starting with the early authorization of a medicine in a restricted patient population, followed by phases of evidence gathering and adaptations of the marketing authorization to expand access to a broader patient population. Adaptive licensing (AL) is based on stepwise learning under conditions of acknowledged uncertainty, with consecutive phases of data collection and regulatory evaluation [44].

It requires the involvement of several stakeholders, including the EMA, the pharmaceutical industry, HTA bodies, and patient organizations. Adaptive licensing builds on existing regulatory processes and intends to extend the use of elements already in place, including scientific advice, compassionate use, the conditional marketing authorization mechanism, patients’ registries, and pharmacovigilance tools, allowing the collection of real-life data and development of risk-management plans.

Initial access to a drug would be limited to a restricted population, defined on the basis of knowledge about risk-benefits at the time of initial approval. Drugs would be labelled as initially authorized, and patients and physicians would be informed of the certainties and uncertainties about the drug. The level of the reimbursement provided to drugs at this level is still an open discussion. Afterwards, the adaptive licensing process would make fuller use of all sources of information, including non-trial patients, to update regulatory and treatment decisions. Evidence generation would not be limited to conventional randomized controlled trials but to a larger methodology spectrum, including trials, clustered randomized controlled trials, observational studies based on medical records, registries, and other forms of surveillance [45, 46].

## Hurdles in clinical practice

### Prescription habits

Once a drug is launched in the market, it takes around 3 years to have maximum impact on survival [2]. This fact is partially attributed to the time needed to diffuse medical information and to change prescription habits. An observation that corroborates this is related to the distribution of drug expenditure: in 2007, two-thirds of the European cancer drug sales were related to drugs approved before 1999 [18].

Three stakeholders participate in this dynamic: the prescribing physician, the pharmacist dispensing the drug, and the patient in need of treatment. Health authorities can control drug costs and prescription by influencing this dynamic. Most EU member states have developed national prescribing guidelines. Guidelines can make suggestions about drug schedules, but also can mandate choices for generic medications. They also target pharmacists by recommending the choice for the cheapest version of a medication, unless there is a clear need for a specific branded product. Monitoring and rewarding physicians that respect a prescription budget or target a percentage of cheaper medicines is a particularly relevant strategy when generic medications become available. The prescription of more expensive drugs can be regulated by the presence of specific conditions, such as predictive or prognostic biomarkers.

### Patient compliance

The final step in the whole process is patient compliance. With cytotoxic intravenous chemotherapy, this factor seems to be less of a problem as these treatments are administered in the hospital setting [47]. However, this information must be interpreted with caution because it derives from patients who agreed to receive intravenous chemotherapy. The proportion of patients declining intravenous chemotherapy is not known.

Compliance is a well-explored topic with respect to oral cancer drugs, varying widely according to the disease setting and the toxicity of the drug [48]. In adjuvant breast cancer trials assessing hormone therapy for more than 4 years, 30–50% of the patients were found to discontinue the treatment prematurely [49]; however, this number can reach up to 80% for haematological malignancies [48] and is largely related to the treatment toxicity. In general, the lack of compliance with respect to oral cancer drugs is positively associated with an increasing toxicity profile and longer treatment periods.

## Conclusions

Future demographic changes in the world’s population will cause cancer to become the largest public health problem worldwide. To face this challenge, improving anticancer drug development and the efficiency of health systems is a top priority. More efficacious anticancer drugs must reach patients in a timely fashion and at affordable prices.

Developing drugs is an increasingly difficult task given the molecular heterogeneity of cancer and the fact that current research models are not yet able to capture this level of complexity. Even after anti-cancer drugs have overcome multiple hurdles in the research process and are finally underway towards regulatory approval, there is a significant remaining challenge.

HTA is becoming progressively important as a mechanism to evaluate the added value of drugs for individuals and for society. They may help governments decide whether to reimburse a given drug, although this entails delays in the routine use of new drugs. When budget restrictions play a major role and cause lengthy delays in cancer drug access, collaboration and the exchange of experiences can help national governments to develop strategies to finance innovative drugs.

This dynamic is influenced by stakeholders with different backgrounds and with complementary expertise. Adaptive licensing in this context seems like a promising approach for accelerating and improving the likelihood of implementation of medical innovation in daily practice. The interaction between researchers, health authorities, national governments, physicians, and patients is a requisite to improving cancer treatment; this will decrease drug lags and bridge the gaps between drug development and its final objective of improving patient care.

## Disclosures

MP: Board member: Pharmaram. Consultant honoraria: Amgem, Astelles, Astra Zeneca, Bayern, Invivis, Lilly, ASD, Novartis, Roche, Genentech, Sanofi-Aventis, Symptogen, Synthos, Verastem. All other authors have declared no relevant conflict of interests.
